# Glutamate and microglia activation as a driver of dendritic apoptosis: a core pathophysiological mechanism to understand schizophrenia

**DOI:** 10.1038/s41398-021-01385-9

**Published:** 2021-05-06

**Authors:** Eduard Parellada, Patricia Gassó

**Affiliations:** 1 Barcelona Clínic Schizophrenia Unit (BCSU). Institute of Neuroscience, Hospital Clínic of Barcelona, University of Barcelona, Barcelona, Catalonia Spain; 2 The August Pi i Sunyer Biomedical Research Institute (IDIBAPS), Barcelona, Catalonia Spain; 3 Centro de Investigación Biomédica en Red de Salud Mental (CIBERSAM), Madrid, Spain; 4 Department of Basic Clinical Practice, Unit of Pharmacology, University of Barcelona, Barcelona, Catalonia Spain

**Keywords:** Schizophrenia, Biomarkers

## Abstract

Schizophrenia disorder remains an unsolved puzzle. However, the integration of recent findings from genetics, molecular biology, neuroimaging, animal models and translational clinical research offers evidence that the synaptic overpruning hypothesis of schizophrenia needs to be reassessed. During a critical period of neurodevelopment and owing to an imbalance of excitatory glutamatergic pyramidal neurons and inhibitory GABAergic interneurons, a regionally-located glutamate storm might occur, triggering excessive dendritic pruning with the activation of local dendritic apoptosis machinery. The apoptotic loss of dendritic spines would be aggravated by microglia activation through a recently described signaling system from complement abnormalities and proteins of the MHC, thus implicating the immune system in schizophrenia. Overpruning of dendritic spines coupled with aberrant synaptic plasticity, an essential function for learning and memory, would lead to brain misconnections and synaptic inefficiency underlying the primary negative symptoms and cognitive deficits of schizophrenia. This driving hypothesis has relevant therapeutic implications, including the importance of pharmacological interventions during the prodromal phase or the transition to psychosis, targeting apoptosis, microglia cells or the glutamate storm. Future research on apoptosis and brain integrity should combine brain imaging, CSF biomarkers, animal models and cell biology.

## Introduction

Schizophrenia is a common and heterogeneous psychiatric disorder that severely affects the lives of roughly 1% of the population worldwide. It is characterized by the presence of positive psychotic symptoms (delusional thinking, hallucinations, and disorganized behavior), negative symptoms (lack of motivation, blunted affect, poverty of expression and social withdrawal) as well as enduring neurocognitive deficits (attention, processing speed, working and long-term memory, executive function, and social cognition). While existing pharmacological treatments are generally able to ameliorate positive symptoms, there has been no way to target the negative and cognitive symptoms despite their clear association with long-term deleterious effects on real-world psychosocial function. For recent reviews see Marder and Cannon^1^, McCutcheon et al.^2^, Owen et al.^3^. Part of the problem is that, in many ways, the neurobiology of schizophrenia remains an unsolved puzzle. To gain a more complete understanding of this disorder, it will be necessary to integrate findings from genetics and epigenetics, environmental factors, molecular biology, neuroimaging, animal models, and translational clinical research.

The neurodevelopmental hypothesis of schizophrenia, in its initial formulation by Weinberger^4^, and Murray and Lewis^5^, or in the “2-hit model” proposed by Keshavan^6,7^, has been the most significant attempt to integrate different findings to explain the origin of this psychiatric disorder. According to this model, early neurodevelopmental insults, like impairment of neurogenesis, neuronal and interneuron migration, dendritic arborization or axonal outgrowth, may lead to abnormalities of specific neural circuits that account for clinical premorbid signs and symptoms, such as mild impairments in behavior, cognition, or social functioning, observed in individuals who later develop schizophrenia. During adolescence and young adulthood, postnatal brain maturation abnormalities like myelination defects, excessive pruning of dendritic spines and loss of synaptic plasticity could account for the emergence of symptoms^8–11^. Furthermore, the dopamine hypothesis formulated by Carlsson and Lindqvist in the sixties^12^, as an explanation for the genesis of positive psychotic symptoms, has given way to the glutamate hypothesis in recent decades. This newer view proposes that glutamatergic dysfunction may play a key role in schizophrenia pathophysiology, particularly regarding negative symptoms and cognitive dysfunction^13,14^.

Fortunately, in the last decade we have had access to the largest genomic study on schizophrenia published to date from the *Schizophrenia Working Group of the Psychiatric Genomics Consortium*^15^. These genetic findings have identified more than 100 loci associated with the risk of schizophrenia which converge into a coherent set of biological processes that align with the predominant etiological hypotheses of the disorder mentioned above^16^. The main genes that have been identified are those encoding synaptic proteins involved in synaptic plasticity, an essential function for learning and memory; genes encoding N-methyl-D-aspartate (NMDA) and α-amino-3-hydroxy-5-methyl-4-isoxazole-propionic acid (AMPA) glutamate receptors, the voltage-dependent calcium channel (VDCC), and dopamine receptor D2 (DRD2); and genetic loci variants in the major histocompatibility complex (MHC) of the immune system^15^. More recently, genetic variations in the complement component 4 (C4) gene have also been identified providing additional evidence of the implication of the immune system in schizophrenia^17,18^. At the same time, repeated neuroimaging studies have revealed gray matter loss as well as abnormal functional connectivity in schizophrenia patients^19–21^. In addition, postmortem studies of patients have reported reduced numbers of dendritic spines, especially on pyramidal neurons located in layer III in the prefrontal cortex (PFC), in the superior temporal gyrus (particularly in the primary auditory cortex), and in certain hippocampal (HPC) subfields^22–26^.

**Fig. 1:**
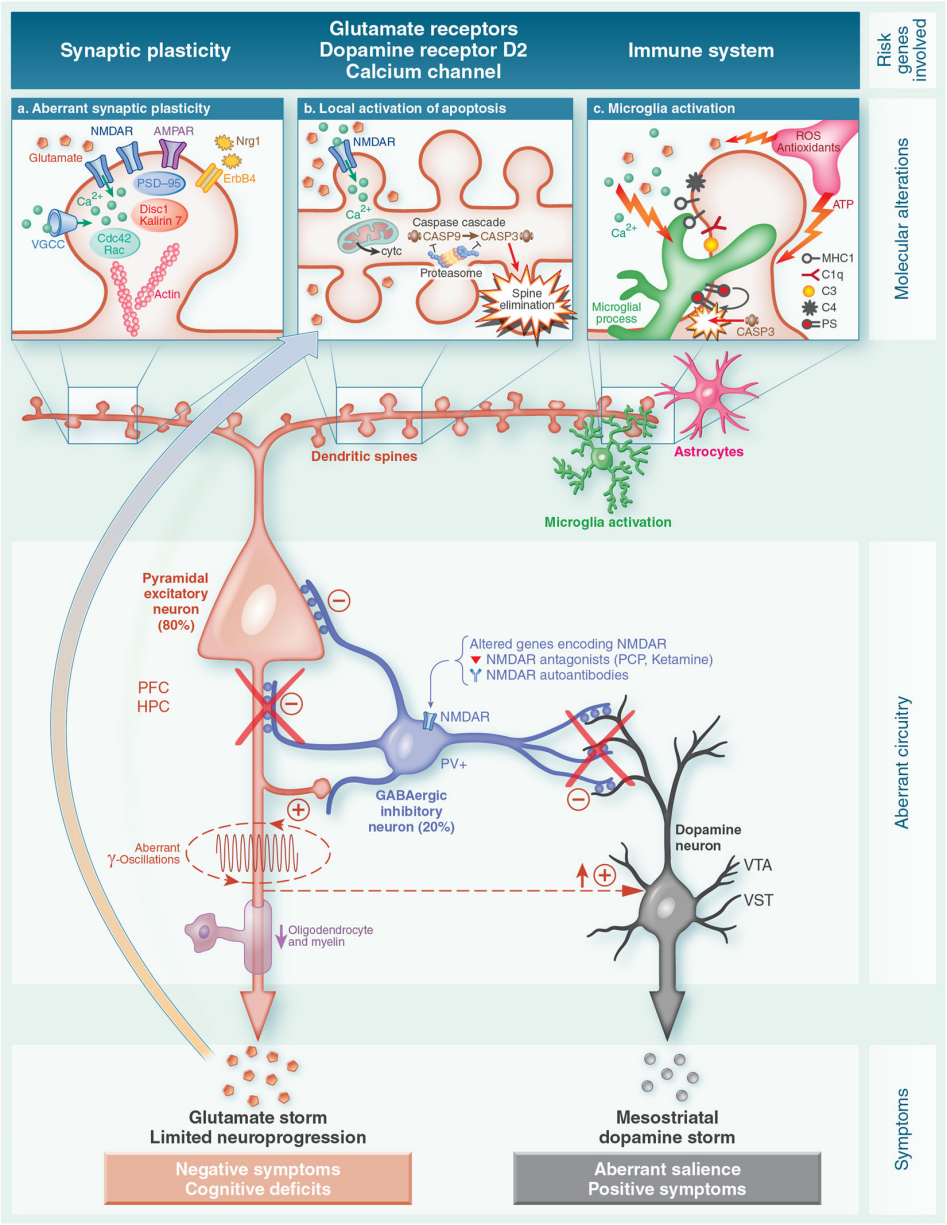
From risk genes to clinical symptoms. Highly simplified diagram summarizing the hypothesized pathways toward dendritic apoptosis underlying the overpruning of dendritic spines occurring in late adolescence and early adulthood in individuals with schizophrenia. The central part of the diagram depicts the alterations reported in brain cortical circuits in individuals with schizophrenia and in animal models of this illness *(Figure modified from Marín)*^44^. Although not represented here separately, it should be noted that inhibitory interneurons (central neuron) are located either in the cerebral cortex (prefrontal cortex –PFC- and hippocampus –HPC-) or in the ventrotegmental area (VTA) and ventral striatum (VST) dopaminergic areas. The altered encoding (among other possible causes) of glutamate N-methyl-D-aspartate receptors (NMDAR) could produce a dysfunction of inhibitory GABAergic interneurons expressing parvalbumin (PV+) resulting in disinhibition of excitatory pyramidal cells (left neuron). Note that there are two translational models supporting this view: anti-NMDAR autoimmune encephalitis and the administration of NMDAR antagonists such as phencyclidine (PCP) and ketamine. The imbalance of excitatory and inhibitory neurons are proposed to lead to aberrant gamma oscillations thereby contributing to the cognitive dysfunction and primary negative symptoms. Abnormalities of oligodendrocytes and myelin have also been described. The dysfunction of GABAergic inhibitory interneurons could produce a regionally-located significant release of glutamate *(glutamate storm*) by excitatory glutamatergic cortical pyramidal neurons as well as a subcortical hyperdopaminergic state *(dopamine storm)*. The elevations in extracellular glutamate might act as a pathogenic driver in the brain triggering apoptosis and limited neuroprogression. On the other hand, the disinhibition of the mesostriatal dopaminergic neurotransmission (right neuron) could cause aberrant salience and either premorbid sub-threshold psychotic symptoms or, if sustained, the full blown-onset of a first psychotic episode. **a** Aberrant synaptic plasticity. Altered genes encoding the fine-tuning of the glutamate synapse which are crucial for spine plasticity and maintenance: NMDAR (*GRIN2A*), α-amino-3-hydroxy-5-methyl-4-isoxazole-propionic acid (AMPA) receptors (*GRIA1*), neuroregulin 1 (NRG1) and its receptor ErbB4, cytoskeletal proteins of the dendritic spines (Actin, ARC complex, RHO, CDC42, Rac, PSD95, DISC1, Kalirin-7). Their dysfunction may contribute to exaggerated spine loss, leading to brain misconnections and synaptic inefficiency *(Figure modified from 0wen and colleagues)*^3^. **b** Local activation of dendritic apoptosis. In critical periods, the glutamate storm and calcium overload via NMDAR could trigger local activation of the dendritic mitochondrial apoptosis pathway and caspase-3 cascade leading to the overpruning of spines and dendrites. Interestingly, altered encoding of voltage-gated calcium channels (VGCC) gene (*CACNA1C*) has been reported and could contribute to making neurons susceptible to calcium overload. As depicted in the figure, proteasomes act as brakes preventing the spread of the apoptosis mechanism to the cell body, thus avoiding cell death (*Figure modified from Ertürk and colleagues)*^87^. **c** Microglia activation. Apoptotic dendrites and other molecules generate “find me” signals and “eat me” signals (e.g.,phosphatidylserine-PS-) to attract microglia contributing to the phagocytosis of synapses. Some candidate molecules such as proteins of the major histocompatibility complex (MHC) class 1 and complement cascade proteins (C1-C3, and, more recently, C4) have also been implicated. Thus, microglia could contribute to the overpruning of dendritic spines in critical periods of neurodevelopment (Figure modified from Miyamoto and colleagues)^97^.

As noted above, excessive dendritic spine pruning during late adolescence and early adulthood may lead to the emergence of symptoms of schizophrenia during these critical periods^8,25,26^. Dendritic spines are protrusions from neuronal dendrites that act as hubs that facilitate excitatory synaptic communication between neurons. In the nineteenth century, the neuroanatomist Ramón y Cajal was the first to describe dendritic spines on neurons^27^. A dendritic spine contains an actin cytoskeleton, NMDA and AMPA glutamate receptors and other proteins regulating synaptic plasticity. From an evolutionary perspective, the appearance of a higher density of dendritic spines in humans led to an increase in our neural synaptic connections, providing a clear advantage that differentiated us from other primates^28^. In individuals with schizophrenia, the loss of dendritic spines may further impair synaptic plasticity (Fig. 1a) which is already impaired by risk gene-sets linked to NMDA or AMPA glutamate receptors or to synaptic proteins underlying the fine-tuning of glutamate synapses. Such gene sets could include, for example, Disrupted-in-Schizophrenia 1/Kalirin-7^29–31^, or the RhoGTPase cell division cycle 42^32^. A loss of cytoskeletal scaffolding proteins in spines could also alter glutamatergic receptor signaling^26^. The loss of dendritic spines and synaptic plasticity would severely alter the micro- and macroconnectivity of cerebral circuitry in individuals with schizophrenia^33^, and impair long-term potentiation (LTP) and long-term depression (LTD), both essential in learning and memory^34^. Long-term potentiation promotes the enlargement of spine heads, whereas LTD leads to spine shrinkage. Glutamate, via NMDAR and AMPAR, is the crucial regulator. However, as discussed below an excess of glutamate can be detrimental to the integrity of dendritic spines. In experimental studies, LTD induction by low-frequency glutamate stimuli resulted in a retraction of dendritic spines. By contrast, LTP induction by repetitive uncaging of glutamate produced a selective enlargement of the spines (small and large) associated with synaptic efficacy. Long-term potentiation is also characterized by the insertion of AMPA receptors into the spine’s surface. Small or thin spines are more dynamic and plastic than large ones and could function as “learning spines”, whereas large spines (mushroom spines) are more stable over time and could be the substrate for memory processes^28,35^. Both cognitive functions are seriously affected in schizophrenia.

Four decades ago, Feinberg was the first to suggest aberrant peri-adolescent pruning of synapses in schizophrenia^36^. Dendritic pruning is a physiological process during neurodevelopment which leads the brain to strengthen some connections and eradicate others through experience-dependent plasticity. It typically overlaps with the period of onset for schizophrenia. However, the molecular mechanisms underlying dendritic pruning are not fully understood even in normal development^37,38^. Dendritic pruning shares several molecular features with apoptosis and it is hypothesized that components of the apoptotic machinery could be involved in this process^39–41^. Recently, it has been reported that microglia activation and molecules of the immune system also contribute to the observed reduction in dendritic spine density in schizophrenia^17,42^. In this article we discuss possible pathways toward dendritic apoptosis, which may well be one of the core pathophysiological mechanisms to help understand schizophrenia.

## NMDA glutamate receptor alterations and the dysfunction of inhibitory GABAergic interneurons in the PFC and HPC (Fig. 1)

According to the glutamate hypothesis of schizophrenia^13,14^, NMDA glutamate receptor alterations, can produce a dysfunction of inhibitory GABAergic interneurons expressing parvalbumin (PV+) resulting in disinhibition of excitatory glutamatergic pyramidal cells (the principal neurons of the cerebral cortex, constituting 80% of all neurons there). The fast-spiking PV + interneurons synchronize the firing of pyramidal neurons underlying the generation of gamma band oscillations, which is critical to optimal cognitive function. Electrophysiological studies in schizophrenia have shown disrupted synchronization of neural gamma oscillations associated with cognitive and negative symptoms^43^. GABAergic interneurons have diverse morphologies (chandelier or basket cells) but are typically aspiny and constitute 20% of all neurons in the cerebral cortex^44^. The PV + interneurons are highly sensitive to NMDA R antagonism as discussed below. They are also vulnerable to oxidative stress, needing a well-regulated antioxidant system to neutralize the overproduction of mitochondria-generated reactive oxygen species^45,46^. Moreover, in mice, it has been found that the deletion of the gene encoding the NR1 subunit of NMDA receptors from PV + interneurons leads to disinhibition of excitatory pyramidal cells and is enough to trigger schizophrenia-like symptoms^47^. Interestingly, in genetically predisposed individuals, cannabis abuse increases the risk of schizophrenia by enhancing GABAergic dysfunction in a subtype of interneurons containing high levels of cannabinoid receptor 1^48,49^. Thus, we can see different paths that converge, leading to the same results.

Although this hypothesis seems much more complex than its initial formulation^50–52^, there are translational models that can still shed light on the issue. The first is anti-NMDA receptor encephalitis, which can be considered a human translational model. This is a synaptic autoimmune disorder in which IgG autoantibodies target NMDARs in the brain (specifically the GluNR1). Patients frequently manifest with prominent psychiatric symptoms, particularly psychosis, early in the disease course^53–55^. It is worth noting that autoantibodies targeting AMPA receptors are prone to lead to limbic encephalitis (confusion, memory impairment and seizures). In a mouse model, the passive cerebroventricular infusion of NMDAR antibodies from patients over a two-week period caused changes in memory and behavior in parallel with an antibody-mediated reduction of synaptic NMDAR^56^. Thus, anti-NMDA receptor encephalitis represents a natural human model of psychosis and supports the glutamate hypothesis of schizophrenia.

A second translational approach to help us better understand schizophrenia, involves NMDAR antagonists (for a historical perspective, see Moghaddam and Krystal)^57^. Animal models have been used to investigate the post-natal administration of NMDA receptor antagonists such as phencyclidine^58–60^, or ketamine^61–63^, which inhibit GABAergic inhibitory interneurons. Ketamine exposure in postnatal mice has been shown to transiently disrupt NMDAR function, produce schizophrenia-like behavioral symptoms in adulthood and persistently induce changes in GABAergic interneurons, including a reduction in PV+ interneurons in the PFC and HPC, particularly in the dentate gyrus^61,64^. Furthermore, it is well known that the use of these glutamate NMDA receptor antagonists mimics the positive, negative and cognitive symptoms of schizophrenia in healthy volunteers and exacerbates psychotic symptoms and cognitive decline in individuals with the disease^65,66^. A recent meta-analysis of post-mortem studies found that PV+ interneurons were reduced in the PFC of patients with schizophrenia^67^. In addition, in an elegant mixed human-animal model, Schobel et al. administered the NMDA antagonist ketamine in mice, confirming the hypothesis that excess glutamate drives hippocampal hypermetabolism and atrophy in psychosis^68^.

The dysfunction of GABAergic inhibitory interneurons expressing PV + could produce both a significant release of glutamate *(glutamate storm*) by excitatory glutamatergic pyramidal neurons in cortical areas and a subcortical hyperdopaminergic state (*dopamine storm*). Both of these phenomena might be due, in part, to lack of braking, although the long-range pyramidal cortical projection to the ventrotegmental area (VTA) could also contribute to the hyperdopaminergic state^69^. As mentioned above, increases in extracellular glutamate might act as a pathogenic driver in the brain^68^, and it is well known that glutamate-gated channels make neuronal cells susceptible to excitotoxicity^39^. On the other hand, the disinhibition of mesostriatal dopaminergic neurotransmission could cause aberrant salience and either premorbid (transient or attenuated) psychotic symptoms or, if sustained, the full-blown onset of a first psychotic episode^70^. In this sense, dopaminergic dysregulation appears to develop downstream of abnormalities in the glutamatergic system and it does not seem to be due to primary dysfunction^71–73^. Although this question goes beyond the scope of this article, it should be noted that increased DA transmission in the mesolimbic-striatal system probably runs in parallel with DA hypoactivity in the PFC.

## Accelerated dendritic pruning around the onset of schizophrenia through local activation of apoptosis **(**Fig. 1b**)**

The glutamate storm and calcium overload, via glutamate receptors, would trigger neurotoxicity and accelerated dendritic pruning through local activation of the mitochondrial apoptosis pathway and caspase-3 cascade, leading to loss of density of dendritic spines. Apoptosis is a form of programmed cell death that is regulated by a complex cascade of pro- apoptotic (Bax) and anti-apoptotic (Bcl-2 and Bcl-XL) proteins in which caspases are the final executors and key drivers of the apoptotic program (for a recent and impressive review about neuronal cell death see Fricker et al.)^39,74^. Apoptosis is characterized by cell shrinkage, membrane blebbing, chromatin condensation, DNA fragmentation, and cellular disintegration with phagocytosis. Another well-known hallmark of apoptosis is phosphatidylserine (PS) externalization in the cell membrane, which is a key signal for the removal of apoptotic cells by surrounding phagocytic cells, such as macrophages and neutrophils. Apoptosis occurs without inflammation and proceeds without a gliosis response. Interestingly, the absence of gliosis is a repeated finding in schizophrenia. Apoptosis is triggered by two principal pathways: the intrinsic (or mitochondrial, via cytochrome c release) pathway and the extrinsic (or external death receptor) pathway. Physiological apoptosis eliminates injured or diseased neurons during lifespan but also during crucial stages of normal brain development (20–80% of all neurons die by apoptosis in early development). Abnormal dendritic apoptosis has also been implicated in the early stages of Alzheimer’s disease. Specifically, the loss of dendritic spines and synapses in the HPC and cortex is an early event in Alzheimer preceding neuronal death^39^. Strangely enough, the increased apoptotic susceptibility could help explain the inverse epidemiological comorbidity that both neurodegenerative diseases and schizophrenia have with cancer. Patients with schizophrenia have a significantly lower risk of lung, prostate and colorectal cancers than do individuals without schizophrenia^75^.

In recent years, it has been proposed that increased susceptibility to apoptosis might be the reason for synaptic or dendritic neuronal loss in individuals with schizophrenia. Two decades ago, the Columbia group was the first to propose this challenging hypothesis^76–79^. Since then, in addition to glutamate excitotoxicity, other pathophysiological processes involved in schizophrenia, such as oxidative stress and lack of neurotrophic factors (particularly, brain-derived neurotrophic factor-BDNF-), have been potentially associated with apoptotic mechanisms^45,46,76,80,81^. Results from our own research group strongly support this hypothesis. We have observed increased apoptotic susceptibility in primary fibroblast cell cultures obtained from a skin biopsy of naïve patients with a first psychotic episode^82^. Using magnetic resonance imaging (MRI) and spectroscopy (1H-MRS), we also found a correlation between altered apoptotic markers and both the volume of certain brain regions and the concentration of glutamate plus glutamine neurometabolites^83^. In a recent microarray study, in which gene expression was analyzed in primary fibroblast cell cultures and in immortalized lymphocytes from the same patients and healthy controls, we also observed alterations in the expression of genes involved in the apoptotic pathways and other important biological functions such as cytoskeleton organization^84^.

As mentioned above, an excess of glutamate could be the trigger of these phenomena of synaptic apoptosis which result in a loss of synapses and dendrites rather than neuronal death (non-lethal synaptic apoptosis)^85^. Generally speaking, cortical volume loss in schizophrenia occurs in the absence of neuronal cell loss and, at least in the PFC, HPC and non-mediodorsal thalamic nucleus, there are no changes in the number of pyramidal neurons^86^. But what is particularly interesting is that it is a local apoptosis that *affects the branches but not the trunk of the tree*. Animal models have shown local caspase activity in neurons in which caspases were confined to the dendritic compartment of pruning neurons and were not detected in the soma or axon^40,41^. Similarly, using an optogenetic approach, Ertürk and colleagues suggested that NMDA receptor activation can trigger this apoptotic pathway locally in dendrites^87^. The local activation of apoptosis through caspase-3 within distal dendrites is enough to prune dendritic spines and branches locally. Apoptosis inhibitory proteins and proteasomes act as brakes to limit the activation and prevent the spread of apoptotic events to the neuronal cell body, thereby preventing cell death.

The reason why dendrites and their spines are lost in some brain regions and not in others is not yet well understood, although it could be related to certain areas having a higher density of inhibitory interneurons and NMDA receptors, such as the dorsolateral PFC and HCP^88,89^. In the animal experiments cited above, NMDA antagonists caused an extraordinary increase in extracellular glutamate in the PFC^14,57^. Interestingly, Schobel and colleagues in their aforementioned study, utilizing an in vivo glutamate biosensor method in multiple hippocampal subfields, found that ketamine causes a regionally preferential increase in extracellular glutamate in, for example, the CA1 and subiculum^68^. We could refer to this as a regionally-located glutamate storm. Looking at the timeline of dendritic spine loss, it is important to note that, in primates, excitatory neurons in layer 3 undergo the most dramatic elimination during the adolescent period^26^. In regard to the timing of apoptosis, it has also been suggested that NMDA receptor-mediated excitotoxicity is more common in young rodents, but not in adults, probably due to loss of caspase-3 expression in neurons with age^39^.

A recent meta-analysis of postmortem studies found lower levels of synaptic proteins, dendritic spines, and GABAergic and glutamatergic markers in patients with schizophrenia^90^. In terms of clinical translational findings, in different brain areas including the PFC, where synapses occupy 6% and their dendrites 30% of gray matter, respectively^86^, the loss of dendritic spines could be related to the accelerated gray matter loss observed in several longitudinal MRI studies, the primary negative symptoms and the cognitive deficits of the disease^21,23^.

Recently, Bossong and colleagues, using 1H-MRS, found an association between increased baseline hippocampal glutamate levels and adverse outcomes in individuals at high risk for psychosis^91^. Using the same technique, in first episode psychosis, higher levels of glutamate in the anterior cingulate cortex were associated with a poor antipsychotic response^92^. Furthermore, the meta-analysis by Marsman and colleagues provides support that there is a progressive decrease of frontal glutamate and glutamine in patients with schizophrenia, possibly indicating a progressive loss of synaptic activity^93^. Taken together, these observations suggest that there is a decrease in glutamate levels after the glutamate storm occurring around the onset of schizophrenia, and that this runs in parallel with the glutamatergic synapse density decrease. We could refer to this as a transient glutamate storm associated with the early stages of schizophrenia.

## Activation of microglia contributes to dendritic apoptosis **(**Fig. 1c**)**

In recent years, microglia cells have attracted a great deal of attention. Microglia are the resident immune cells of the central nervous system, performing an essential function during neuroinflammation, while also playing a role in noninflammatory processes like axonal guidance, neurotrophic support, natural apoptosis of developing neurons and synaptic pruning^94,95^. Once considered mere passive sentinels of immunity, microglia now seem to be crucial for pruning back dendrites during neurodevelopment, particularly in adolescence^95,96^. During dendritic apoptosis, caspases and other molecules, such as phosphatidylserine (PS), generate signals to attract microglia for rapid removal of synaptic debris. Thus, synaptic pruning involves microglial phagocytosis of synapses^39,97,98^.

During the developmental refinement of neural circuits in adolescence, there is an increase in the density of resident brain microglia cells for synaptic pruning/remodeling. The different timing of synaptic pruning in different cortical regions likely reflects differences in the onset of the critical periods of circuit refinement^99^. It is not fully understood how microglia determine which synapses to prune and which to avoid, however it is known that microglial contact with apoptotic dendrites increases with higher electrical activity (calcium flux). Moreover, looking at analogies with the peripheral immune system, several elegant animal studies have shown that apoptotic dendrites release “find me” signals (e.g., ATP release from astrocytes, levels of glutamate, calcium overload, etc.) and “eat me” signals (e.g., phosphatidylserine or proteins of the MHC class I family as well as complement cascade proteins of the immune system, including C1q and C3)^39,96,98,100–103^. These findings are in accordance with the hypothesis presented here, combining the glutamate theory of schizophrenia and microglial synaptic pruning. At the same time, another class of immune molecules commonly called “don’t eat me” signals are essential to counterbalance the effects of “eat me” signals (e.g., the transmembrane immunoglobulin CD47). This molecule protects synapses from excess microglia-mediated pruning during neurodevelopment^104^. Interestingly, recent genome-wide association studies have identified relevant risk loci for schizophrenia including the MHC and the complement C4^17^. Thus, complement abnormalities and proteins of the MHC via microglia activation may contribute to the increase of dendritic apoptosis leading to an excess of synaptic pruning in schizophrenia. Recently, Sellgren and colleagues, using isolated synaptosomes based on patient-derived induced microglia-like cells (iMG), demonstrated increased synapse elimination that could be partly explained by schizophrenia risk variants at the complement C4 locus. They also showed that the antibiotic minocycline reduces microglia-mediated synapse uptake in vitro. Moreover, its clinical use was associated with a decrease, although modest, in the incidence of schizophrenia risk compared to other antibiotics in young adults identified using an electronic health record^42^. However, these preliminary results need confirmation in prospective randomized clinical trials. Besides cell models, positron emission tomography (PET) imaging could be another promising tool to evaluate in vivo microglial activation. It is assumed that when microglia cells are activated, this increases the expression of the 18-kDa translocator protein (TSPO) which can be measured with PET radiotracers. However, it is still not clear whether TSPO expression is specifically linked to microglia^105^. A recent meta-analysis of in vivo microglial PET imaging studies in individuals with schizophrenia gave inconclusive results, but further studies are needed with more specific PET radioligands^106,107^.

Taken together, the points discussed above seem to confirm that there is a noninflammatory role of the microglial activation. In recent years, elevated levels of some proinflammatory cytokines have been attributed to putative “inflammation” of the brain. However, this conclusion overlooks the fact that cytokines can also have noninflammatory roles related to plasticity, immunity, or brain homeostasis. Thus, although activated microglia and elevations of cytokines are often cited as a marker of “neuroinflammation”, we consider this term unfortunate when used to describe schizophrenia or other psychiatric disorders (for a further discussion of this issue see Estes and McAllister)^81,94,108,109^.

## Therapeutic and research implications

Current pharmacological treatments mainly consist of antipsychotic drugs blocking post-synaptic dopamine D2 receptors. This is effective for positive psychotic symptoms but has little effect on negative and cognitive symptoms. Future drugs should focus on aberrant neurodevelopment and disease progression^110^. To achieve this, several approaches could be further explored, especially at early stages of disease, targeting aberrant synaptic plasticity, the glutamate storm, dendritic apoptosis, calcium channel dysfunction and microglia activation (Table 1):

**Table 1:** Hypothetical link between the risk genes and proteins involved in schizophrenia and their pathophysiology, clinical outcomes and both ongoing and future treatments currently under investigation.

Risk genes /proteins involved	Pathophysiology	Clinical implications	Outcome	Therapeutic implications
SYNAPTIC PLASTICITY (PSD95, ARC complex, actin and other cytoskeletal proteins of the dendritic spines)	Aberrant circuitry LTD and LTP failure for learning and memory Inability to form new synapses	Negative and cognitive symptoms	Negative	Drugs promoting the formation of dendritic spines and preventing their loss (intranasal peptide davunetide –AL-108-; estrogens such as raloxifene; clozapine?)
NMDA (*GRIN2A*) and AMPA (*GRIA1*) GLUTAMATE RECEPTORS	Disrupted excitatory-inhibitory balance Regionally-located glutamate storm-apoptosis and synaptic overpruning in PFC and HPC	Disrupted brain gamma-oscillations contributing to negative and cognitive symptoms Gray matter loss (CT, MRI) High levels of glutamate (1H-MRS) associated with poor outcome and treatment resistance	Negative	Modulators of GABAergic system Attenuation of glutamate release by inhibitors of VGSC (Evenamide (NW-3509A)) AMPA Modulators (Ampakine CX516) Anti-glutamatergic drugs modulating metabotropic mGlu2/3 receptor Antiapoptotic effects of SGAPs? Inhibitors of caspase-3 (Q-VD-OPh; Z-VAD-fmk)* Blockers of cytochrome c release of the mitochondrial apoptotic pathway*
VDCC (*CACNA1C*)	Calcium overload-apoptosis and synaptic overpruning	Gray matter loss (CT, MRI)	Negative	Calcium channel antagonists?
IMMUNE SYSTEM (MHC and proteins of the complement system: C1q,C3,C4)	Contributes to apoptosis and synaptic overpruning via microglial activation	Gray matter loss (CT, MRI)	Negative	Drugs targeting microglia activation such as minocycline or other drugs blocking microglial phagocytic receptors
*DRD2*	Contributes to the downstream mesostriatal dopamine dysregulation	Aberrant salience Positive psychotic symptoms (sub-threshold in prodromal stage; first-episode psychosis and relapses) Increased dopamine transmission (PET, SPECT) Dopamine overreactivity after amphetamine administration	Positive/Negative (TRS)	Current antipsychotic drugs blocking dopamine D2 receptors New drugs regulating dopamine function (synthesis, vesicular storage, dopamine D2 autoreceptors)**

*AMPA: alfa-amino-3-hydroxy-5-methyl-4-isoxazole-propionic acid; ARC: activity-regulated cytoskeleton-associated protein; CACNA1C: calcium voltage-gated channel subunit alpha 1C; CT: computerized tomography; DRD2: dopamine receptor D2; GABA: gamma-aminobutyric acid; HPC: hippocampus; LTD: Long-term depression; LTP: Long-term potentiation; MCH: major histocompatibility complex; MRI: magnetic resonance imaging; NMDA: N-methy-D-aspartate; PET: positron emission tomography; PFC: prefrontal cortex; PSD: post-synaptic density protein; SGAPs: second-generation antipsychotics; SPECT: single photon emission computerized tomography; TRS: treatment-resistant schizophrenia; VDCC: voltage-dependent calcium channel; VGSC: voltage-gated sodium channel; 1H-MRS: magnetic resonance spectroscopy*.

*Only investigated in preclinical studies in animal models of some classic neurodegenerative disorders.

**For a recent review see Kaar and colleagues^131^*.*

(1) Promoting dendritic spines and preventing their loss in individuals who are at high risk for the illness during the prodromal or transitional phase of psychosis has been proposed as a key target in the future treatment of schizophrenia^25,26,86,87^. The intranasal peptide davunetide (AL-108), acting on microtubule polymerization in neurons, has shown preliminary effects improving memory and cognition in mice. Nevertheless, its efficacy in ongoing clinical trials awaits corroboration^111^. Raloxifene, a selective estrogen receptor modulator, can increase in vitro spine density in the PFC and improve cognitive symptoms when administered in healthy postmenopausal women and in schizophrenia patients, although results are inconsistent^112^. Some antipsychotic drugs, such as clozapine, the gold-standard treatment for treatment-resistant schizophrenia, promote the formation of dendritic spines, increasing some of the post-synaptic density proteins, such as spinophilin or shank1a puncta. Moreover, compared to haloperidol, clozapine increased the growth of the spines while changing their shape to be more like mushroom spines^113^. Finally, it should be mentioned that microRNAs (miRNAs), a class of nonprotein-coding RNAs, could serve as drugs for medical intervention. One example would be miRNAs that modulate gene-protein signaling pathways involved in the polymerization of F-actin filaments of the dendritic spine cytoskeleton. Inappropriate levels of mature miRNA result in the reduction of synaptic proteins, compromising the structure and functionality of the dendritic spines^114^;

(2) Modulation of the upstream glutamate and GABA systems that regulate dopamine firing could be another approach. In an animal model, a pharmacological modulation of the hippocampal GABAergic system has been reported^115^. Another potential therapeutic target to help correct dysfunctional GABAergic interneuron signaling is the alpha 5 subtype of the GABAA receptor, which is highly expressed in the human amygdala and HCP^116^. However, its potential efficacy remains to be seen. Evenamide (NW-3509), a selective inhibitor of voltage-gated sodium channels which attenuates glutamate release, is under trial as an adjunctive treatment for resistant schizophrenia^117^. Ionotropic AMPA glutamate receptors, which are involved in LTP and synaptic plasticity, could be another focus, although the ampakine CX-516 has been found not to be effective for improving cognitive symptoms in individuals with schizophrenia^118^. To prevent the glutamate storm, further research is needed with anti-glutamatergic drugs such as those that modulate type 2/3 metabotropic glutamate receptors (mGlu2/3), which are expressed on presynaptic nerve terminals and widely distributed in the brain, and which inhibit the presynaptic release of glutamate^63,119,120^. Some of these, such as LY354740^63^, LY2140023^121^, or JNJ40411813, the positive allosteric modulator (PAM) of the mGlu2 receptor^122,123^, may have failed to treat established cases of schizophrenia, but it could be helpful to investigate their possible benefits during critical early periods of the disease such as the prodromal stage or during the transition to psychosis. Along these lines, we have recently found that treatment of adult mice with JNJ-46356479, a new PAM of the mGlu2 receptor^124^, partially improves neuropathological deficits and schizophrenia-like behaviors in a postnatal ketamine mice model^64^, and we are currently investigating the administration of this PAM in adolescent mice. Impeding the glutamate storm may be particularly effective during early stages of schizophrenia to prevent or slow the progression of the illness according to the disease modification hypothesis^68,110^;

(3) Owing to the plausible relevance of dendritic apoptosis in the pathophysiology of schizophrenia, a challenging approach that remains to be investigated involves trying to prevent dendrite removal with the inhibitors of caspase-3 or other components of the mitochondrial apoptosis pathway^39,40,87^. We must bear in mind that modulation of apoptosis demands great caution, especially if such treatments are applied in the early phases of schizophrenia. It is well known that in some cell types and tissues, excessive apoptosis could induce atrophy, while a reduced rate of apoptosis may lead to uncontrolled cell proliferation such as cancer. In preclinical studies, inhibitors of caspase-3 such as Q-VD-OPh and Z-VAD-fmk have been explored in some classic neurodegenerative disorders^39^. Another focus could be blockers of cytochrome c release in the mitochondrial apoptotic pathway. Such blockers have been found to prevent dopaminergic neuronal death by apoptosis in a rat model of Parkinson’s disease^125^. It is also worth mentioning here that certain antipsychotics could have a neuroprotective role in vitro through an antiapoptotic action, as was shown in a previous study from our group using a neuroblastoma cell model^126^. A more recent study also found that clozapine may have a protective/anti-apoptotic effect on adult neural stem cells from ketamine-induced cell death in correlation with decreased apoptosis and autophagy^127^;

(4) VDCC, found in dendrites and dendritic spines of cortical neurons, could be another potential target, although no evidence has yet been found of the efficacy of any typical calcium channel antagonist such as gabapentin or pregabalin^128^;

(5) Specific pharmacological interventions targeting microglial elimination of synapses should also be examined, particularly those preventing aberrant synaptic pruning which occurs in the early stages of schizophrenia. These could include the tetracycline antibiotic minocycline or blockers of the microglial phagocytic receptors^39,42,129^. Minocycline, as reported earlier, has been found to reduce microglia-mediated synapse uptake in a schizophrenia patient-derived model of synaptic pruning^42^. Moreover, twelve months of add-on minocycline in recent-onset schizophrenia seems to protect from gray matter loss in the fronto-temporal cortical regions^129^, although its potential clinical efficacy remains to be established in clinical trials. Another approach could be blockers of the microglial phagocytic receptors (e.g.,vitronectin receptor, Mertk) that recognize “eat me” signals (e.g., phosphatidylserine or complement factors exposed on the surface of apoptotic dendritic spines) or “don’t eat me” signals (e.g., cell surface immunoglobulin CD47 localized on synapses)^39,104^. Again, caution is required when manipulating the microglial function because it is fairly well-established that microglia have both inflammatory and non-inflammatory physiological roles^130^.

(6) Finally, as depicted in Table 1, it has long been known that genes encoding DRD2 are also implicated in schizophrenia and new drugs that target the regulation of the dopamine system are under investigation. However, this topic is beyond the scope of our paper and we refer the reader to the recent publication by Kaar et al.^131^.

Peripheral biomarkers do not fully capture the disease process which primarily occurs in the brain^132^. This is why a concerted effort should be made to examine central cerebrospinal fluid (CSF) biomarkers of dendritic pruning in both first-episode psychosis as well as in longitudinal studies^133^. Given the heterogeneity of the illness, stratification of individuals according to future clinical outcomes is crucial. Neurogranin, which is expressed exclusively in the brain, particularly in dendritic spines, seems to be a promising novel CSF biomarker for synaptic loss^134,135^. Another potential biomarker may be levels of kynurenic acid, which is an endogenous antagonist of the NMDA glycine modulatory site^136,137^. Other approaches will come from new genetic animal models and optogenetic studies in rodents^33,69^. Of particular interest are knockout mutant mice that have genes knocked out of microglia cells but no other cells. In animal models, refined imaging techniques such as intravital Multiphoton Microscopy now provide the opportunity for high resolution detection of microglia cells in the living brain^138^. Finally, another approach that could be investigated is human cellular models, by applying cellular reprogramming methods to create patient-specific in vitro models of dendritic pruning^42^.

Last but not least, since psychotic relapses, especially those occurring in the early stages, have been associated with gray matter loss, treatment resistance and poor outcome, it would be interesting to evaluate what happens during a psychotic relapse in terms of brain integrity^139–141^. The findings, in turn, will allow us to clarify a long-standing hypothesis, not yet proven, suggesting that active psychosis could be neurotoxic^142^. To accomplish this aim in vivo, we need to explore central CSF biomarkers, find more specific PET radioligands with reliable binding at the NMDA receptor, or identify PET ligands for in vivo evaluation of apoptosis phenomena or microglial activation^106^. It was thought that labeled annexin-V as PET radiotracer, could detect in vivo externalized phosphatidylserine (PS), one hallmark of apoptosis, but this approach did not live up to its promise. Imaging cell death by apoptosis remains an unsolved problem in clinical molecular imaging, and efforts are needed to overcome this limitation^143^. One promising approach that is currently under investigation involves using caspase-based specific PET radiotracers to detect caspase-3 activation^144^. Regarding 1H-MRS, further studies are needed using more sophisticated functional MRS protocols to evaluate brain glutamate levels and discern whether observed increases are due to increased neurotransmission rather than metabolism, since it is difficult to disentangle the peaks from glutamate and glutamine^145^. A combination of brain imaging, especially PET, 1H-MRS, CSF biomarkers, and cell biology may help improve our understanding of these relevant clinical issues.

In conclusion, in this article we have highlighted some of the pathways toward dendritic apoptosis that could stem from a transient glutamate storm and might be aggravated by microglial activation. This links the glutamate hypothesis with the intervention of the immune system in the etiology of schizophrenia. These phenomena could explain the exaggerated synaptic pruning that occurs during late adolescence or early adulthood which might be a core feature underlying schizophrenia. Of course, many other crucial questions remain, such as the timeline of events, the exact nature and location of the cells and neural circuits affected, as well as details about possible cause-effect relationships (e.g., which is the primary event, a neuronal excitation imbalance or a primary microglia dysfunction?). Additionally, it should be remembered that the mechanisms mentioned here may be neither exclusive to schizophrenia nor representative of all types of the disease, given its great heterogeneity^16,133^. At the same time, considering that schizophrenia is a multifactorial and highly polygenic disorder that shares many risk genes with other psychiatric illnesses including bipolar disorder, depression, intellectual disability and autism spectrum disorders, it is to be expected that these diseases may also share some of these same mechanisms which, in each case, may disrupt different key brain circuits. This driving hypothesis has relevant therapeutic implications. Specifically, it suggests that pharmacological interventions during critical periods of the disease, such as the prodromal stage or during the transition to psychosis, should target dendritic apoptosis, microglia or preventing the glutamate storm. Future research on dendritic apoptosis, brain integrity and clinical outcomes should combine brain imaging techniques, central CSF biomarkers, animal models and cell biology.
